# Magnetic resonance imaging for acute appendicitis in pregnancy: can clinical scores predict when imaging is needed?

**DOI:** 10.1007/s00068-024-02538-3

**Published:** 2024-05-16

**Authors:** Noam Kahana, Elad Boaz, Mariya Neymark, Hayim Gilshtein, Yossi Freier Dror, Ofer Benjaminov, Petachia Reissman, James Tankel

**Affiliations:** 1 Department of General Surgery, Shaare Zedek medical center, the Hebrew University School of Medicine, Jerusalem, Israel; 2 Department of General Surgery, Rambam Health Care Center, Haifa, Israel; 3 Mashav Applied Research, Jerusalem, Israel; 4 Department of Radiology, Shaare Zedek Medical Center, the Hebrew University School of Medicine, Jerusalem, Israel

**Keywords:** Pregnancy, Acute appendicitis, MRI, Diagnosis, Clinical scoring

## Abstract

**Purpose:**

Advanced imaging may augment the diagnostic milieux for presumed acute appendicitis (AA) during pregnancy, however it is not clear when such imaging modalities are indicated. The aim of this study was to assess the sensitivity and specificity of clinical scoring systems with the findings on magnetic resonance imaging (MRI) of AA in pregnant patients.

**Methods:**

A retrospective cohort study between 2019 and 2021 was performed in two tertiary level centers. Pregnant patients presenting with suspected AA and non-diagnostic trans-abdominal ultrasound who underwent MRI as part of their evaluation were identified. Patient demographics, parity, gestation, presenting signs, and symptoms were documented. The Alvarado and Appendicitis Inflammatory Response (AIR) score for each patient were calculated and correlated with clinical and MRI findings. Univariate analysis was used to identify factors associated with AA on MRI.

**Results:**

Of the 255 pregnant patients who underwent MRI, 33 (13%) had findings of AA. On univariate analysis, presentation during the second/third trimester, migration of pain, vomiting and RLQ tenderness correlated with MRI findings of AA. Whilst 5/77 (6.5%) of patients with an Alvarado score ≤4 had signs of AA on MRI, a score of ≥5 had a sensitivity, specificity, negative and positive predictive value of 84.8%, 36.6%, 94.0% and 17.2%. For an AIR score ≥ 5, this was 78.8%, 41.5%, 93.0%, and 16.7%, respectively.

**Conclusions:**

Whilst clinical scoring systems may be useful in identifying which pregnant patients require MRI to be performed when AA is suspected, the low sensitivity implies further research is needed to refine the use of this valuable resource.

## Introduction

Recent global level evidence reports the age standardized incidence and prevalence of acute appendicitis (AA) to be 8.7 and 229.9/100,000 population with a 30% increase in the past 30 years [1]. AA is the most common cause of non-gynecologic emergency surgery among pregnant patients, with reported incidence rate of 1:1250-1:1500 [2]. Nevertheless, the diagnosis of AA in pregnancy is challenging as the typical clinical signs of AA are also common during pregnancy, blood tests may be misleading and the displacement of the appendix by the enlarged uterus make clinical evaluation difficult [3, 4]. Considering these challenges, radiological adjuncts can be used to ensure a timely and accurate diagnosis of AA as a delayed diagnosis increases the risk of perforation and has been associated with early delivery and fetal loss [5, 6]. Conversely, unnecessary surgery has been associated with small for gestational age, lower birth weight and earlier delivery [5, 7].

Abdominal US has a sensitivity of 50–78% in diagnosing AA during pregnancy becoming less efficacious with advanced gestational age and increased maternal BMI [8–11]. Magnetic Resonance Imaging (MRI) is the next preferred test as this modality avoids the ionizing radiation of computed tomography (CT) and has a reported sensitivity and specificity of up to 97% and 99% [12–14]. However, it is not clear which patients would benefit from this study being performed following a non-diagnostic abdominal ultrasound. Being able to stratify patients according to the likelihood of an MRI adding to the diagnostic milieux is important not only because the long-term safety of MRI in pregnancy has not yet been definitively demonstrated [15], but also because it is an expensive investigation often limited by lack of access [16].

Among pregnant patients, the Alvarado and Appendicitis Inflammatory Response (AIR) scores have been shown to have a sensitivity of 86.36% and 74.24%, and specificity of 61.54% and 76.92% respectively with surgically confirmed AA [17]. Considering data that has correlated these scores with the likelihood of AA being revealed on abdominal CT [18, 19], a similar approach could be used to stratify patients for whom MRI would be helpful in achieving a diagnosis.

However, no such studies have been performed in the setting of pregnant patients with regards to MRI. We hypothesized that clinical scores could help identify pregnant patients in whom MRI may reveal AA following a non-diagnostic abdominal ultrasound. The aim of this study was to explore sensitivity and specificity of clinical scoring systems with the findings of AA on MRI performed in pregnant patients.

## Methods

### Study setting

We performed a retrospective cohort study based in two urban, high-volume, university affiliated, tertiary level hospitals in Jerusalem and Haifa, Israel. Between these two centers, there are approximately 30,000 live births per year and 750 appendectomies performed, of whom 30 are performed in pregnant patients. Local ethics approval was sought from the ethics committee of each institution with the requirement for informed consent waived (0220-21-RMB-D and 0109-19-SZMC).

### Patient selection

Patients were identified via a search of the electronic database of the MRI departments at each hospital. These databases keep a record, maintained in real-time, of basic clinical and demographic data for each patient undergoing an MRI. Between the 1st of January 2019 and 31st of December 2022, all female patients of child-bearing age who underwent an MRI of the abdomen and pelvis were identified. These records were then manually reviewed and the studies performed due to a suspicion AA were extracted. The electronic and paper medical records of these patients were then retrieved and the patients were then verified according to the following inclusion criteria. Pregnancy must have been confirmed with a raised serum beta HCG and/or a transabdominal/transvaginal ultrasound revealing an intrauterine pregnancy, have been seen emergently in the department of Emergency Medicine (ED) or acute gynaecology assessment unit with routine blood tests taken on presentation to the hospital. Clinical assessment could have been performed by either a member of the gynecology, emergency or general surgery teams. A non-diagnostic transabdominal ultrasound must have been performed by a senior radiology trainee or board certified attending in radiology. A study was defined non diagnostic if it the appendix was not visualized or another cause of the patient’s presenting complaint identified. Finally, the MRI must have been performed for presumed diagnosis of AA and have been reported by an attending radiologist.

### Data extraction

The primary aim of the study was to explore the relationship between two commonly used clinical scoring systems with the incidence of AA on MRI performed during pregnancy. In order to achieve this aim, using the patient’s electronic medical records augmented by paper charts the following data was extracted.

The Alvarado and AIR scores for AA were calculated as previously described [20, 21]. The elements of these scores are summarized in Table 1. The results of the MRI findings, operative reports and pathological diagnoses were extracted. A normal appendix was defined as the absence of gross inflammation, AA was defined as the presence of inflammatory changes of the appendix, and complicated AA referred to those specimens in which perforation or gangrene was present.

Patients were allocated to one of two groups (AA on MRI and no AA on MRI) depending on the radiological findings on MRI and a univariate analysis performed comparing clinical and biochemical variables.

### Imaging protocol and analysis

All MRI examinations were performed on a 1.5– T system (Siemens, Erlangen, Germany). Breath Hold Axial T1 VIBE Dixon (TR 133, TE 5/5, Flip Angle 75), T2 HASTE in 3 planes (3350, 108, 160), T2 HASTE fat-suppression (3350, 108, 160), T2 TRUFI coronal and axial (525, 2, 60) sequences were obtained using a body phased array coil (18 elements). Slice Thickness of 4 mm with a gap of 0.5mm was used. The field of view (FOV) was chosen to be as small as possible. The T1W sequences were used to assess the adrenal glands for the possibility of Adrenal Infarct. No sedation or anesthesia was used. The average MRI examination was completed within 20 minutes. All MRI exams were read on our Picture Archiving and Communication System (PACS, Centricity, GE Healthcare) and finalized by body imaging fellowship trained Radiologists.

AA was defined radiologically as dilatation of the appendix > 6 mm, edema of the appendiceal wall, peri-appendiceal fat-stranding or fluid and/or the presence of a peri-appendiceal abscess.

### Statistical analysis

The data was recorded in Microsoft Excel™ (Microsoft Corporation, Washington) and statistical analysis performed using SPSS™ (version 27, SPSS Inc, Chicago, Illinois, USA). The demographic and clinicopathological data are presented as mean or frequency with standard deviation or percentages in parenthesis respectively. For univariate analysis, continuous variables were compared using students T test whilst categorical variables were analysed using either Chi squared or Fishers exact test as appropriate. A receiver operator curve (ROC) was used to identify the ideal cut-off for sensitivity and specificity for the calculated Alvarado and AIR scores. A p value of < 0.05 was considered statistically significant.

## Results

During the study period, a total of 255 pregnant patients underwent MRI for suspected AA. Among them, 33 patients (13%) had MRI findings consistent with AA, whilst 222 patients (87%) did not. A univariate analysis comparing the demographic, clinical and biochemical variables of the cohort stratified by MRI findings is shown in Table 2. When stratified by MRI findings, demographic and obstetric was comparable between the the two groups in terms of patient age, BMI and gestational age at time of diagnosis.

The physical findings associated with AA on MRI included the migration of abdominal pain to the RLQ (10/33, 30.3% vs. 27/222, 12.2%, *p* = 0.006) and vomiting (15/33, 45.5% vs. 35/222, 15.8%, p = < 0.001). There was a trend towards the presence of RLQ tenderness on examination and anorexia being associated with AA on MRI. With regards to the biochemical values, the percentage neutrophilia was higher among patients with an MRI that showed signs of AA (79.5%, ± 6.8 vs. 75.1%, ± 7.9, *p* = 0.004). Conversely, the mean leukocytosis, neutrophil-to-lymphocyte ratio and level of C-reactive protein did not differ according to MRI findings.

Once the Alvarado and AIR score were calculated for each patient, a comparison of the sensitivity, specificity, positive predictive value (PPV) and negative predictive value (NPV) was calculated according to the MRI findings. Among the patients with an Alvarado score ≤ 4, 5 out of 77 (6.5%) had MRI findings in keeping with AA. As shown in Table 3, among those with an Alvarado score of ≥ 5 or higher, the sensitivity, specificity, NPV and PPV for the MRI showing signs of AA were 84.8%, 36.6%, 94.0%, and 17.2%, respectively. Similarly, Among the patients with an AIR score ≤ 4 or lower, 7/99 (7.1%) had MRI findings of AA. In contrast, an AIR score of ≥ 5 had a sensitivity, specificity, NPV and PPV of 78.8%, 41.5%, 93.0%, and 16.7%, respectively, for signs of AA on MRI. The area under the curve of the receiver operator curve for both the Alvarado and AIR score was 0.600.

An ad hoc analysis was performed using these cut-offs in terms of the likelihood of having AA on MRI across different trimesters. As shown in Table 4 and no relationship was found between the different trimesters and the MRI findings.

Out of these 33 patients with AA on MRI, 29 underwent laparoscopic appendectomy. AA was seen clinically in 28 cases including 3 patients with complicated AA, 2 of whom had periappendicular abscesses and one who had a peri-appendicular phlegmon. The remaining 4 patients were successfully treated with antibiotics alone, none of whom had findings of complicated AA on MRI. All patients with complicated appendicitis on MRI had this finding confirmed pathologically. There was one patient who had radiological findings in keeping with AA in whom a macroscopically normal appendix was seen at surgery. However, uncomplicated AA was confirmed pathologically in all those with MRI finding in keeping with this diagnosis. No patient with an MRI that revealed a normal appendix underwent appendectomy or were treated with empirical antibiotics.

## Discussion

In this study, we explored the relationship between examination findings, biochemical variables and clinical scores in pregnant patients with signs of AA on MRI following a non-diagnostic abdominal ultrasound. We found that whilst subtle clinical findings were associated with AA on MRI, Alvarado and AIR scores of ≥ 5 had a high sensitivity but low specificity for AA.

Pregnancy presents a diagnostic and therapeutic challenge when AA is suspected. As noted in the wide-ranging review by Gorter et al., no surgical approach can be recommended over another however a multidisciplinary approach involving surgeons, anesthetists and obstetricians should be involved to ensure the best maternal and fetal outcomes [22]. Appendectomy performed in the setting of a normal appendix is associated with lower neonatal birthweights and a higher risk of small for gestational age infants (OR 5.6, 95% CI 1.02–30.9) [7]. At the same time, a timely and accurate diagnosis of AA during pregnancy is crucial to avoid the complications associated with a delayed diagnosis.

Transabdominal US is often used as a first line investigation in the diagnosis of AA. However, in a recent meta-analysis, the sensitivity of US in the first, second and third trimester was 69%, 63% and 51% respectively [23]. This is reflected in the data presented above as only 9% of patients with AA on MRI were in their first trimester highlighting the greater diagnostic yield of transabdominal US earlier in pregnancy. There also seems to be a stepwise relationship between increasing BMI and the decreasing likelihood of visualization of the appendix on abdominal US performed during pregnancy for a suspected AA [24]. The limitations of US later in pregnancy and in patients with a higher BMI has given rise to the option of using cross-sectional imaging to help diagnose AA in those patients for whom abdominal US is equivocal and diagnostic uncertainty remains.

We found that 13% of the pregnant patients who underwent MRI had findings consistent with AA with a sensitivity of 100% confirmed by intraoperative and pathological findings. These results are comparable to previous studies and highlighting the value of MRI in diagnosing AA during pregnancy [8, 12, 13, 20]. Whilst this may suggest that many studies are being unnecessarily performed, we argue that the low sensitivity but high specificity highlights the power of MRI in helping the remaining patients potentially avoided unnecessary treatment for an incorrectly diagnosed AA. This is of particular clinical interest as routine blood tests did not correlate with the incidence of AA further strengthening the notion of the power of MRI in directing patient care.

Despite the power of MRI in diagnosing AA, efforts to identify the patients who would benefit from this expensive and limited modality are under-reported in the academic literature. A single report of 29 pregnant patients in whom only 2 had AA found no association between the Alvarado score and MRI findings [25]. In the study by Gentles et al. of 164 pregnant patients, AA was more likely to be found on MRI if there was a history of emesis, migratory pain, rebound tenderness, raised white cell count and neutrophilia and a mean Alvarado score of 6.5 [26]. However, in this study ultrasound was not routinely used as a first line investigation, limiting the generalizability of the findings to the many centers where access to MRI is more limited.

Interestingly, a subset of patients with AA were treated successfully with antibiotics, avoiding the need for surgery. This observation suggests that a non-operative approach can be considered in selected cases with MRI findings consistent with AA. Importantly, prospective data describing the non-operative management of AA did not include pregnant patients among the study cohort [27]. Nevertheless, retrospective data from Japan found that among 113 pregnant patients with AA treated non-operatively, including 6% with complicated AA, fetal and maternal outcomes were similar when compared to those treated surgically [28]. Conversely, population level data found that non-operative management of complicated AA failed in 32.7% of patients with delayed surgery associated with preterm delivery, labor and abortion [29]. Taken together, this suggests that whilst uncomplicated AA can likely be managed non-surgically, early operative intervention is likely warranted in pregnant patients with complicated AA due to the high failure rate of non-surgical management. However, the finding that 12% of patients suffer from recurrence of appendicitis during the same pregnancy has led major guidelines to support surgical intervention in all pregnant patients with AA until those at risk of failure of non-operative intervention can be identified [30].

Our univariate analysis revealed a significant correlation between migration of pain towards the RLQ, vomiting and a leftward neutrophil shift with MRI findings consistent with AA. These clinical symptoms have been traditionally associated with AA and serve as important indicators in the diagnostic evaluation. Despite the difficulty in diagnosing AA in pregnancy, we suggest that the presence of these symptoms should raise the suspicion of AA in pregnant patients, prompting further investigation and the consideration of performing an MRI if an US is inconclusive. Such an approach is also supported by recent international guidelines [30]. It is interesting that, in comparison to previous reports, leukocytosis, neutrophil-lymphocyte ratio and C-reactive protein did not correlate with MRI findings of AA suggesting a limited discriminatory power for differentiating the value of performing an MRI for AA in pregnant patients based on these parameters [2, 20]. This finding might be related to the relatively low prevalence of AA in our report.

The diagnostic performance of the Alvarado and AIR scores was assessed in this study. Whilst other clinical scores have been found to be more accurate in diagnosing AA in pregnancy [31], limitations in the ability to collect the data to compute these scores precluded their inclusion. Despite this, the findings described above suggest that while both scoring systems have moderate sensitivity and good NPV, they are limited in terms of specificity and PPV. This reflects the limitations of the physical examination and biochemical findings described above. As a result, it is likely that many MRI scans performed for suspected AA will continue to be negative. Our center has previously described the safety of a stepwise approach to managing AA during pregnancy with laparoscopic appendectomy performed only if there was clinical or biochemical deterioration following a non-diagnostic US [32]. It may well be that considering the low specificity of MRI for a presumed AA during pregnancy, a more judicious approach could consist serial observations in the setting of a low Alvarado or AIR score.

The novelty and strength of this study is that it is the first to correlate clinical scoring systems with radiological findings on MRI performed for an AA during pregnancy. By demonstrating the relationship between clinical scores, we highlight a potential threshold that can be used for selecting which patients may ultimately require imaging particularly when access to the resource is limited.

It is important to acknowledge the limitations of our study. Firstly, this was a retrospective study with a relatively small sample size that may limit the generalizability of our findings and accuracy of the data collected. Further limitations in the data collection also precluded the calculation of other clinical scores. Additionally, the retrospective nature of the study and the potential for selection bias should be considered when interpreting the results as those patients with a low suspicion of AA who did not undergo MRI were not captured in the study. We were also unable to include the time since presentation to hospital and the time the MRI was performed as a metric for analysis. Considering the small number of patients with complicated AA, it was also not possible to perform a subgroup analysis with regards to the sensitivity and specificity of the scores among these specific patients. Finally, the differences between the patients who did, and did not have, AA on MRI affects the generalizability of the results described.

In conclusion, our study highlights that among pregnant patients with a suspected diagnosis of AA in whom transabdominal ultrasound is non-diagnostic, an Alvarado or AIR score of ≥5 could be used as a threshold for performing an MRI scan for diagnostic confirmation. These findings emphasize the importance of integrating clinical symptoms and scoring systems with the decision to perform advanced cross-sectional imaging to optimize the diagnosis of AA during pregnancy. However, the low pre-test probability highlights the need to use these scores as adjuncts to clinical decision making. Diagnostic algorithms need to be refined in order to improve the specificity of MRI in the diagnostic milieux of pregnant patients in whom AA is suspected.

**Table 1:** Clinical scores for acute appendicitis

Alvarado score	
Variable	Score
Migratory right lower quadrant pain	1
Anorexia	1
Nausea or vomiting	1
Tenderness in the RLQ	2
Rebound tenderness in the RLQ	1
Fever > 37.5 °C (> 99.5 °F)	1
Leukocytosis of white blood cell count > 10 × 109/liter	2
Total	9
Appendicitis inflammatory response (AIR) score	
Vomiting	1
RLQ pain	1
Rebound tenderness (Light/Medium/Strong)	1/2/3
Fever > 38.5 °C (> 101.3 °F)	1
Polymorphonuclear leukocytes < 70%/70–84%/>85%	0/1/2
Total	8

**Table 2:** A comparison of examination findings, biochemicals variables and operative findings stratified by MRI findings

Variable	AA on MRI	No AA on MRI	*P* value
	*N* / mean (% / SD)	*N* / mean (% / SD)	
	*N* = 33	*N* = 222	
Age (years)	28.5 (± 5.7)	28.7 (± 5.4)	0.943
BMI (kg/m^2^)	26.8 (± 2.7)	26.0 (± 3.3)	0.745
Parity	2.8 (± 1.6)	2.2 (± 1.6)	0.587
**Trimester**			
1st	3 (9.1%)	51 (22.9%)	
2nd	23 (69.7%)	119 (53.7%)	
3rd	7 (21.2%)	52 (23.4%)	0.135
RLQ pain	21 (9.5%)	204 (91.9%)	0.222
Migration of the pain towards the RLQ	10 (30.3%)	27 (12.2%)	0.006
Vomiting	15 (45.5%)	35 (15.8%)	< 0.001
Anorexia	5 (15.2%)	14 (6.3%)	0.07
**Fever**			
> 37.0	25 (75.8%)	162 (73.6%)	0.823
> 38.5	25 (75.8%)	158 (71.8%)	0.643
Urinary symptoms	0 (0.0%)	2 (6.1%)	0.564
RLQ tenderness	32 (97.0%)	188 (84.7%)	0.056
Biochemical variables:			
Leukocytosis (> 10mm^3^)	22 (66.7%)	154 (69.4%)	0.835
Neutrophilia (%)	79.5 (± 6.9)	75.1 (± 7.9)	0.004
Neutrophil to leukocyte ratio	6.7 (± 4.7)	5.6 (± 5.3)	0.631
C-reactive protein	4.1 (± 5.5)	4.5 (7±.0)	0.573
Non-diagnostic ultrasound	33 (100.0%)	222 (100.0%)	> 0.999
**MRI findings**			
Acute appendicitis	30 (90.9%)	-	
Complicated acute appendicitis	3 (9.1%)	-	-
**Operative findings**			
Macroscopically normal appendix	1 (3.0%)	-	
Acute appendicitis	25 (75.8%)	-	
Complicated appendicitis	3 (9.1%)	-	
Treated non-surgically	4 (12.1%)	-	-
**Pathological findings**			
Acute appendicitis	26 (78.8%)	-	
Complicated appendicitis	3 (9.1%)	-	-

**Table 3:** Efficacy of Alvarado and AIR score ≥ 5

Score	NPV	PPV	Sensitivity	Specificity
Alvarado score	94.0%	17.2%	84.8%	36.6%
AIR score	93.0%	16.7%	78.8%	41.5%

**Table 4:** Incidence of acute appendicitis on MRI stratified by Alvarado and AIR scores and trimester

Trimester	Alvarado score ≥ 5	Alvarado score < 5	*P* vaue
1	3/38	0/16	
2	18/91	5/51	
3	7/35	0/24	0.229
	AIS score ≥ 5	AIS score < 5	
1	2/28	1/26	
2	18/92	5/50	
3	6/34	1/25	0.304
